# Microvascular Response to the Roos Test Has Excellent Feasibility and Good Reliability in Patients With Suspected Thoracic Outlet Syndrome

**DOI:** 10.3389/fphys.2019.00136

**Published:** 2019-02-21

**Authors:** Samir Henni, Jeanne Hersant, Myriam Ammi, Fatima-Ezzahra Mortaki, Jean Picquet, Mathieu Feuilloy, Pierre Abraham

**Affiliations:** ^1^Vascular Medicine, University Hospital Center, Angers, France; ^2^UMR INSERM 1083 – CNRS 6015, Mitovasc Institute, Angers, France; ^3^Department of Cardiovascular and Thoracic Surgery, University Hospital Center, Angers, France; ^4^Sports Medicine, University Hospital Center, Angers, France; ^5^LAUM, UMR CNRS 6613, Groupe ESEO, Angers, France

**Keywords:** peripheral artery disease, exercise, provocative maneuvers, ischemia, microcirculation, oximetry

## Abstract

**Background:** Exercise oximetry allows operator-independent recordings of microvascular blood flow impairments during exercise and can be used during upper arm provocative maneuvers.

**Objective:** To study the test-retest reliability of upper-limb oximetry during the Roos test in patients with suspected thoracic outlet syndrome (TOS).

**Materials and Methods:** Forty-two patients (28 men, 14 women; mean age: 40.8 years) were examined via transcutaneous oxygen pressure (TcpO2) recordings during two consecutive Roos tests in the standing position. The minimal decrease from rest of oxygen pressure (DROPmin) value was recorded after each maneuver was performed on both arms. The area under the receiver operating characteristic (ROC) curve defined the DROPmin diagnostic performance in the presence of symptoms during the tests. The Mann–Whitney *U*-test was used to compare the DROPmin in the symptomatic vs. asymptomatic arms. The test-retest reliability was analyzed with Bland-Altman representations. The results are presented as means ± standard deviations (SD) or medians [25–75 percentiles].

**Results:** The symptoms by history were different from the symptoms expressed during the Roos maneuvers in one-third of the patients. The DROPmin measurements were −19 [−36; −7] mmHg and −8 [−16; −5] mmHg in the symptomatic (*n* = 108) and asymptomatic (*n* = 60) arms, respectively. When TOS observed on ultrasound imaging was the endpoint, the area under the ROC curve (AUC) was 0.725 ± 0.058, with an optimal cutoff point of −15 mmHg. This value provided 67% sensitivity and 78% specificity for the presence TOS via ultrasound. When symptoms occurring during the test represented the endpoint, the AUC was 0.698 ± 0.04, with a cutoff point of −10 mmHg. This provided 62% sensitivity and 66% specificity for the presence of pain in the ipsilateral arm during the test. The test-retest reliability of DROPmin proved to be good but not perfect, partly because of unreliability of the provocation maneuvers.

**Conclusion:** To the best of our knowledge, this study is the first to investigate microvascular responses during the Roos maneuver in patients with suspected TOS. The presence of symptoms was significantly associated with ischemia. TcpO2 facilitated the recording of both macrovascular and microvascular responses to the Roos test. The Roos maneuver should probably be performed at least twice in patients with suspected TOS.

## Introduction

Thoracic outlet syndrome (TOS) is one of the most controversial disorders in medicine, with multiple recent editorials having been published on the subject (Ahmad and Murthy, 2018; Burt, 2018; Illig, 2018). TOS comprises a variety of symptoms and physical findings resulting from the external compression of vessels and/or nerves as they pass through multiple narrow spaces between the thorax and the upper limb (Jubbal et al., 2018; Kaplan et al., 2018). As described by Demondion et al. (2006), the diagnosis is based on clinical findings, but making this diagnosis is often difficult. The use of imaging (radiography, ultrasonography, computed tomographic angiography, or magnetic resonance imagery) is required to determine the nature and location of the underlying structures causing the compression (Ammi et al., 2017).

Many provocative maneuvers have been proposed to induce dynamic compression of the neural or vascular structures. A provocative test was proposed by Roos in 1966, which is referred to as the “Roos test” or the “elevated arm stress test” (EAST). Beyond the reproduction of symptoms induced by the Roos test, an evaluation of perfusion via Doppler, pulse palpation, and/or observation of hand pallor are generally performed to detect arterial compression. However, coldness and color changes could also result from sympathetic vasoconstriction during neurogenic TOS (Sanders et al., 2007). In evaluating vasoconstrictive responses to adrenergic stimulation, the study of skin micro-circulation during provocative maneuvers could be of major interest in the study of TOS, at least for research purposes. To the best of our knowledge, such microvascular investigations have never been reported.

We aimed to evaluate the effects of the Roos (EAST) test on upper arm microcirculation using transcutaneous oxygen pressure (TcpO2) measurements. We hypothesized that this technique would be feasible and could account for both macrovascular and microvascular impairments. We also aimed at estimating the reliability of the responses observed in both unaffected patients and patients referred for suspected TOS. Last, we aimed to identify the preliminary results of preoperative versus postoperative evaluations.

## Materials and Methods

From March 2018 to September 2018, 42 patients were recruited into this prospective single-center study.

### Ethical Standards

The patients were fully informed about the study and its procedures; all patients signed a written consent document. This research and all its procedures were performed in compliance with the principles outlined in the Declaration of Helsinki. The study was promoted by the University Hospital in Angers, approved by our institutional Ethics Committee, and was registered in ClinicalTrials.gov under Ref: NCT03355274.

### Experimental Design

The study population comprised patients referred to the Department of Vascular Medicine for suspected TOS. All subjects were over 18 years of age and there was no maximum age limit set. Exclusion criteria were: pregnancy, any legal constraint, or current participation in another clinical trial. To date, one female patient was referred to us for a new evaluation after a prior surgery. Results from this observation are provided to illustrate the presence or absence of persistent ischemia in the surgically treated arm.

### Initial Assessment

At the point of study inclusion, we recorded patient demographics and conditions including: age, sex, presence of one or more cardiovascular risk factors (hypertension, diabetes mellitus, dyslipidemia, active smoking), history of chest or shoulder surgery, and any ongoing treatments. We also measured weight, height, systolic and diastolic blood pressures, and evaluated positional microvascular responses to the Roos test using transcutaneous oximetry (TcpO2) as described below. Results from ultrasound investigations performed prior to referral were encoded as “presence of absence of positive TO” on each side, according to the patient’s medical record. The technicians or physicians doing the TcpO2 test were blinded to these data.

### Transcutaneous Oxygen Pressure Recordings

**FIGURE 1:**
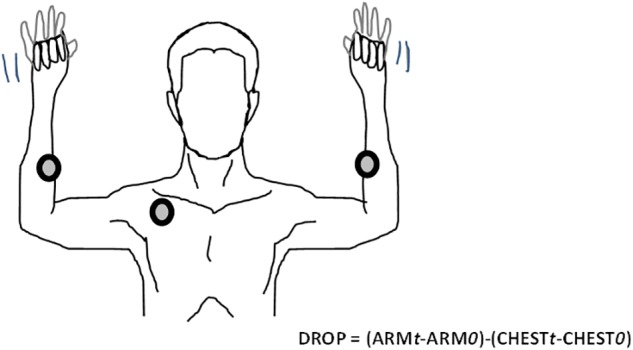
Example of the Roos maneuver with TcpO2 recordings. The DROP calculation at time (t) is based on subtraction of the chest measurement from the arm measurements. Chest and arm changes are accounted for in the mean values measured over the 30 first seconds of recording “0.” TcpO2, transcutaneous oxygen pressure; DROP, decrease from the rest of oxygen pressure.

In brief, TcpO2 is a useful technique that measures the local skin oxygen partial pressure using electrochemical probes heated to 44.5°C to improve local perfusion and oxygen transcutaneous diffusion. TcpO2 changes, by calculating reductions in the rest of oxygen pressure (DROP) index, provide evidence of stimulation-induced regional blood flow impairments (Abraham et al., 2003, 2005; Bouye et al., 2004; Abraham, 2006; Henni et al., 2018). DROP is calculated as the limb level changes minus the changes at a reference chest electrode. DROP allows for removal of error due to unpredictable transcutaneous gradients (Abraham et al., 2003; Grouiller et al., 2006; Blake et al., 2018; Henni et al., 2018). In the present study, we positioned one probe 5–7 cm distal to the elbow joint at the proximal and internal portion of each forearm and one probe on the chest (parasternal) for reference. After a minimum of 15 min of heating, we started a 1-Hz recording with a 30-s reference period, after which patients performed two consecutive Roos tests. The second test was started after the DROP values returned to zero and after a minimum recovery period of 1 min following the end of the first test. Provocative maneuvers were conducted in the standing position with the patient’s back against a wall. The patients were required to raise their arms to 90 degrees of abduction, with the arms fully externally rotated and the elbows at 90 degrees of flexion. Further, we asked the patients to try to touch the wall behind them with their elbows and wrists, but not their back, to ensure that the arms were flexed slightly behind the frontal plane, as shown in Figure 1. This “surrender” or “candlestick” position with opening and closing of both hands was sustained in all patients to the point of maximum pain, or for a minimum of 2 min in the absence of pain. Patients were repeatedly asked to report any symptoms (pain, fatigue, numbness, or tingling) experienced during the Roos test on each arm. The minimum value of DROP (DROPmin) during or in the minute following the end of each Roos maneuver was recorded for each arm. Inherently, DROP is a negative value that decreases with the increase in the severity of ischemia (Abraham et al., 2005) and is expressed in millimeters of mercury (mmHg).

### Statistical Analysis

The data are presented as numbers (percentages), medians [25, 75 percentiles], or means ± standard deviations (SD). A comparison of the DROPmin results between the symptomatic and asymptomatic arms was performed using the Wilcoxon rank test. A receiver operating characteristic (ROC) curve analysis comparing the DROPmin value to the presence/absence of symptoms was used to define the optimal cutoff point for the DROPmin values in the prediction of symptom occurrence. All statistical analyses were performed using SPSS (IBM SPSS statistics V15.0, Chicago, IL, United States). *P* < 0.05 was considered to be statistically significant. The area under the ROC curve (AUC) was used to determine whether DROP significantly predicted positive results on ultrasound imaging and/or the presence of symptoms during the Roos test. The optimal cutoff point was defined as the DROP value that resulted in the minimum distance to the 100% sensitivity / 100% specificity angle. The test-retest reliability of the DROP results observed for two consecutive Roos tests was analyzed using a Bland-Altman representation with logarithmic (10-log) transform in cases of heteroscedasticity, according to recommendations (Euser et al., 2008; Ludbrook, 2010). To create the 10-log transformation, DROPmin values were converted into absolute values (positive values). The absence of a decrease in DROP was encoded by −1 mmHg and not zero. Last, the test-retest reliability of TcpO2 was evaluated according to the similarity of symptoms between test 1 and test 2. Indeed, if discomfort or pain was present during one of the maneuvers, but absent during the other one, the TcpO2 difference was expected to have resulted from unsatisfactory maneuver reproduction, with the presence of compression during one test and the absence of compression during the other.

## Results

The recruited patients included 28 men and 14 women, aged 40.8 ± 12.2 years old. The mean weight was 70.3 ± 16 kg and mean height was 164 ± 7 cm. Fifteen patients had one or more cardiovascular risk factors and 12 had a prior history of chest or shoulder surgery. Ongoing treatments included the use of lipid-lowering drugs (*n* = 2) and sartans (*n* = 2). Systolic and diastolic arm blood pressures were 126 ± 14 and 79 ± 11 mmHg, respectively. Among these patients, 15 reported unilateral symptoms on the right (*n* = 7) or left (*n* = 8) side and 27 reported bilateral symptoms. Among the patients reporting unilateral symptoms, 5 had previously undergone surgery for contralateral TOS. Among patients with unilateral symptoms, Roos maneuvers induced unilateral (*n* = 8) or bilateral (*n* = 5) pain. In two patients, the Roos maneuver did not reproduce the usual symptoms. Interestingly, among patients with unilateral symptoms, when the results were analyzed arm by arm, the symptoms were present in two consecutive tests in 8 patients, in neither of the tests in 5, and in one test only in 17 (in solely the first test in 8, and in solely the second test in 9). Of the 27 patients referred for bilateral symptoms, 2 had undergone previous unilateral (*n* = 1) or bilateral (*n* = 1) surgery for TOS. Among these patients, the Roos maneuvers induced unilateral (*n* = 6) or bilateral (*n* = 20) pain. In one patient, the Roos maneuvers did not reproduce the usual symptoms. Notably, when the 54 arms of the patients with bilateral symptoms were analyzed arm by arm, the symptoms were present during two consecutive tests in 31 patients, during neither test in 10, during solely the first test in 8, and during solely the second test in 5. Of 42 patients, ultrasounds were positive for TOS on one or both sides in only 25 of them, as shown in Table 1.

**Table 1:** Distribution of symptoms by history and results of ultrasound investigations in the studied population.

		Positive ultrasound results			
		None	Right	Left	Bilateral
Symptoms by history	Right	2	3	0	2
	Left	3	2	3	0
	Bilateral	12	2	4	9

**FIGURE 2:**
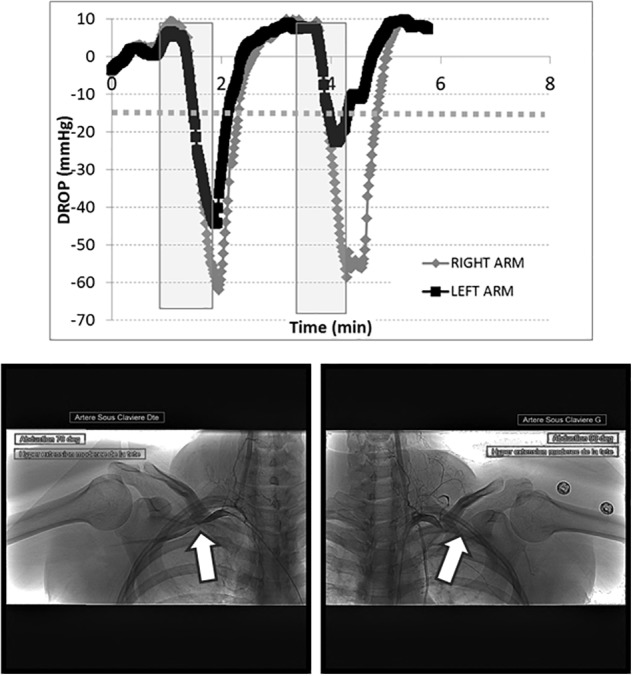
Transcutaneous oxygen pressure recordings are expressed as DROP values (mmHg) during two consecutive Roos maneuvers (gray squares). The *x*-axis represents the time in minutes. As shown, a dramatic decrease in the DROP values occurs during the Roos maneuvers on both sides, below –15 mmHg (dotted line). The corresponding arteriography shows the right (left panel) and left (right panel) sides, confirming occlusion of the subclavian artery during abduction of the arms (white arrows). This arteriography is from a 31-year-old woman who suffered for more than 4 years from upper limb pain, mostly on the right (dominant) side. She previously worked as a hairdresser, but had to stop this activity because of the pain. The pain originated at the shoulder and extended to the hands, with some paresthesias in the fourth and fifth digits. During the clinical examination, the Wright and Adson positions quickly led to a loss of the radial pulses. The Tinel test was negative. Bilateral TOS was suspected. A routine chest X-ray showed no abnormality of the cervico-thoracic junction. A duplex-scan demonstrated dynamic arterial and venous obstructions. Objective signs of lower plexus nerve lesions on electromyogram favored a neurogenic-type outlet syndrome. We also performed a dynamic bilateral angiography via a 4-Fr femoral puncture. This confirmed arterial outlet syndrome with an occlusive costoclavicular grip and a significant dynamic stenosis in the pectoralis minor muscle tunnel. She was prescribed an extended period of kinesiotherapy. DROP, decrease from the rest of oxygen pressure; TOS, thoracic outlet syndrome.

TcpO2 showed excellent feasibility, with no technical failures or missing values. The chest TcpO2 value at rest was 69 ± 10 mmHg. TcpO2 at rest in the symptomatic (69 values) and asymptomatic (15 values) arms were 76 ± 12 and 74 ± 10 mmHg, respectively (*p* > 0.05). A typical example of a recording in a patient with TOS is presented in Figure 2. As shown, the Roos maneuvers were responsible for a sharp decrease in the right and left DROP values, although they were slightly decreased during the second maneuver on the left arm.

The lowest DROPmin values of the two tests were as follows: −12 [−21, −7] mmHg in the arm with an absence of TOS findings on ultrasound imaging and −27 [−50, −14] mmHg in the arms with ultrasound evidence of TOS (*p* < 0.01). When TOS identified on ultrasound imaging was the endpoint, the AUC was 0.725 ± 0.058 (*p* < 0.001 from random choice), with an optimal cutoff point of −15 mmHg providing 67% sensitivity and 78% specificity for the presence of TOS on ultrasound.

On average, the DROPmin values were as follows: −14 [−26, −6] mmHg for test 1 and −13 [−27, −7] mmHg for test 2 (*p* > 0.05) on the right side; −13 [−27, −7] mmHg for test and −11 [−29, −6] mmHg for test 2 on the left side. On an arm-by-arm basis, among the 168 available values (42 patients, 2 arms, 2 tests), the DROPmin values measured in the symptomatic (108 values) and asymptomatic (60 values) arms were −19 [−36, −7] mmHg and −8 [−16, −5] mmHg, respectively (*p* < 0.001). When the presence of symptoms during the test was the endpoint, the AUC was 0.698 ± 0.04 (*p* < 0.001 from random choice), with a cutoff point of −10 mmHg providing 62% sensitivity and 66% specificity for the presence of pain in the ipsilateral arm during the test.

**FIGURE 3:**
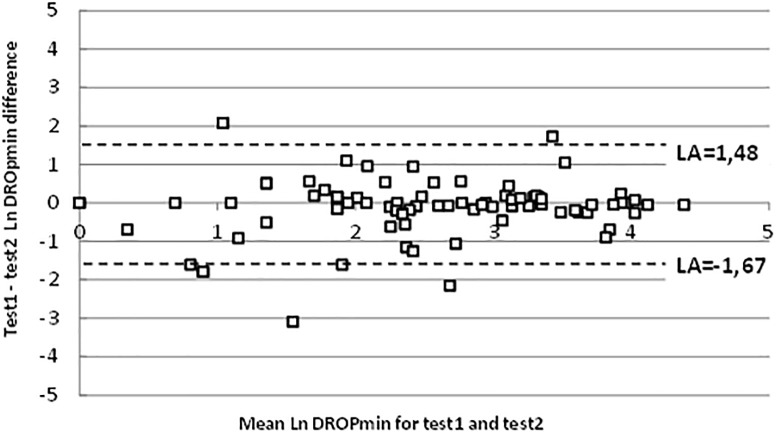
Bland-Altman representation of test-retest reliability on logarithmic scales. “LA” are 95% limits of agreement.

For the entire test series, the average difference between the DROPmin results of test 2 vs. test 1 on a Bland-Altman representation showed a clear heteroscedastic distribution with a mean difference of 1.2 mmHg. Figure 3 shows the 10-log transformed data, confirming good agreement between the two tests (despite outlier values), with a mean difference close to zero and reasonable limits of agreements (LA).

**FIGURE 4:**
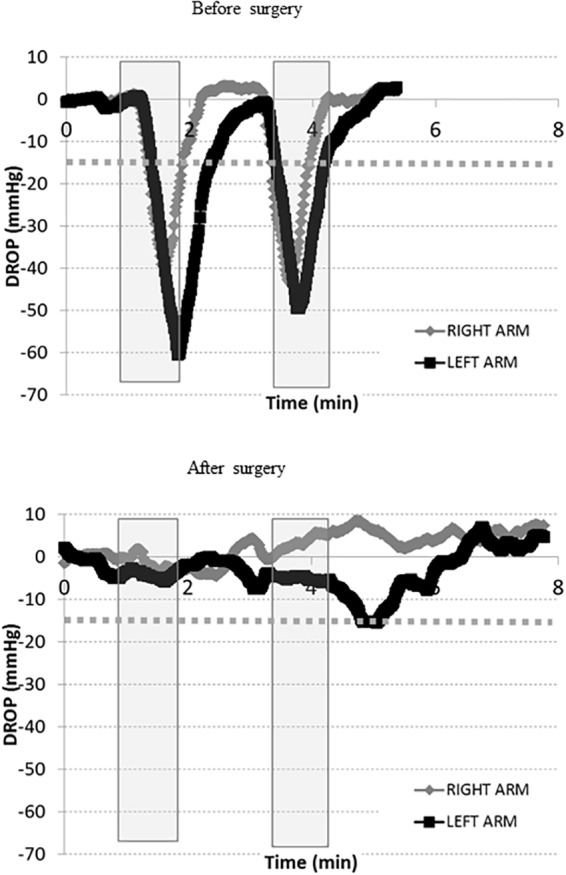
Transcutaneous oxygen pressure recordings expressed as DROP values (mmHg) during two consecutive Roos maneuvers (gray squares). The *x*-axis represents time in minutes before and after surgery. The –15 mmHg limit is shown as a dotted line. These recordings were performed on the same patient described in Figure 2 above. While she previously underwent an extended period of kinesiotherapy for several months without additional benefit, we proposed surgical treatment for her outlet syndrome. As shown, during the new pre-surgical visit (upper panel), the tests are positive on both sides. The surgery included complete resection of the right first rib (image 2) and pectoralis minor muscle resection through a Roos axillary approach. The patient rapidly recovered from surgery and expressed rapid pain relief. After surgery (lower panel), a moderate decrease in DROP is observed with test 2 on the left side, while test 1 was normal on both sides. The patient had no complaints of pain on the right side, but expressed moderate discomfort on the left side with test 2. DROP, decrease from the rest of oxygen pressure.

Finally, the patient presented in Figure 2 had a second test prior to surgery (Figure 4, upper panel), confirming the results of the first test, and then had surgery on the right (dominant) side by an axillary approach. She was referred 3 months after surgery with complete relief of symptoms in the right arm and normalization of the DROPmin on the right side (Figure 4, lower panel). Because her left arm was only moderately symptomatic after surgery, she was unwilling to undergo a second operation.

## Discussion

To the best of our knowledge, this study is the first to investigate microvascular responses during the Roos test, one of the most largely used provocative maneuvers in the diagnosis of TOS. This study provides evidence that the technique is both feasible and reliable. Further, preliminary results suggest that it is sensitive to the changes observed after surgery.

Positive tests suggesting the presence of arterial, and/or venous, and /or nerve compressions are frequently observed in the general population. Using photo-plethysmography (PPG) in 130 apparently unaffected individuals, arterial obstructions were identified in 78 of them (60%) (Gergoudis and Barnes, 1980). Abnormal ultrasound or arterial PPG findings on the EAST and Upper Limb Tension Test (ULTT) were observed in 69% of 64 high-performance string instrument musicians, in contrast to 15% of 22 control subjects (Adam et al., 2018). Arterial flow anomalies on Doppler ultrasound and PPG were identified in 13% of 100 limbs from 50 normal volunteers (Chen et al., 2014). In 98 dissections of the thoracic outlet from 50 unselected cadavers, Juvonen et al. (1995) observed 63% with abnormal anatomy and 10% (5/50) had bilaterally normal anatomy. Thus, most neurovascular compressions remain asymptomatic and the specificity of tests will never reach 100% if symptoms are used as gold-standard endpoints.

There are conflicting results about the respective prevalences of arterial (ATOS), venous (VTOS), and neurogenic (NTOS) types of TOS and “combined” / “disputed” types (Ferrante and Ferrante, 2017; Peek et al., 2018). Relative ATOS, VTOS, and NTOS prevalences seem to partially rely on how each form is defined. Restricting arterial TOS to embolic events and venous TOS to Paget-Schroetter thromboembolic complications, as proposed by Sanders et al. (2007), leads to the consideration that over 95% of all TOS are neurogenic and less than 1% are arterial. Other series have reported more balanced results, using less restrictive definitions for ATOS and VTOS with results ranging from 48.7 to 74% of NTOS, 22 to 44% VTOS, and 4 to 25% ATOS (Ann Freischlag, 2018; Henry et al., 2018; Likes et al., 2014). Finally, whatever the definitions used and the exact proportions of each TOS type, the diagnosis of compression has generally been based on non-invasive clinical signs and vascular investigations.

Clinical provocative tests are considered to be of limited sensitivity and specificity (Warrens and Heaton, 1987; Gillard et al., 2001), probably partially resulting from a high rate of positive results in apparently asymptomatic patients, as previously discussed (Nord et al., 2008); however, it may also possibly result from a low test-retest reliability of the provocative maneuvers (Warrens and Heaton, 1987; Nord et al., 2008). No single non-invasive technique seems to be optimal for diagnosing TOS. One of the most widely used and useful tests for diagnosing TOS is ultrasonography. Unfortunately, preoperative duplex ultrasounds are only 41% sensitive for the diagnosis of NTO (Orlando et al., 2016). Ultrasonography only has a sensitivity of 87% and specificity of 88%, with risks of false-positive and false-negative results (Gillard et al., 2001). False-positive tests are assumed to result from signal losses or, when compared to the presence of symptoms by history, from the possibility of compression in asymptomatic – assumed healthy – individuals. False-negative tests could result either from non-adapted maneuvers, insufficient practitioner experience, or inadequate technical recording. Magnetic resonance imaging (MRI) sensitivity, specificity, and positive and negative predictive values have been reported to be 41, 33, 89, and 4%, respectively (Singh et al., 2014). Whatever the technique, assuming that coldness and color changes would not be caused by ischemia resulting from subclavian artery obstruction, but from an overactive sympathetic nervous system (Sanders et al., 2007), Doppler ultrasonography would not adequately detect neurally induced cutaneous vasoconstriction. Whether or not pallor and coldness are always of neural origin, the specific benefit of TcpO2 is that it can monitor both macro- and microvascular responses according to TcpO2 decreases at arm level. These responses reflect cutaneous flow changes from the eventual occlusion of proximal vessels and the eventual reduction in skin blood flow secondary to sympathetic nervous system-induced vasoconstriction. The specific interest in TcpO2 is that it is expected to decrease in cases of arterial compression (Babilas et al., 2008), secondary to neurally induced vasoconstriction (Provenzano et al., 2008) and isolated venous occlusion (Ostergren et al., 1983; Geis et al., 2008).

To the best of our knowledge, no other research has investigated upper limb TcpO2 during provocative tests to evaluate TOS. Transcutaneous oxygen pressure changes have been well-described at rest and during exercise (Abraham et al., 2003). During exercise, this measurement was shown to be both accurate and reliable in the evaluation of exercise-induced ischemia (Abraham et al., 2003, 2005; Bouye et al., 2004; Abraham, 2006; Henni et al., 2018). Although absolute TcpO2 has shown fair reliability (Rooke and Osmundson, 1989; Daviet et al., 2004), the calculation of the DROP index improved reliability in test–retest recordings (Bouye et al., 2004). In the present study, TcpO2 facilitated the measurement of regional blood flow impairments, regardless of the underlying mechanism, and showed satisfactory reliability. Importantly, the −15 mmHg cutoff point, using ultrasound results as a reference, is similar to the one identified from exercise TcpO2 measurements (Abraham et al., 2003; Grouiller et al., 2006; Audonnet et al., 2017; Henni et al., 2018). Determining the cutoff point is important because limb elevation physiologically decreases TcpO2 as hydrostatic pressure decreases (Dooley et al., 1997; Shah et al., 2008).

The present study had some limitations. First, we did not monitor the positions of the patients using three-dimensional cameras or accelerometers. This would be useful for excluding the ∼15% inconsistent test-retest results that relied on an imperfect reproducibility of carefully performed provocative maneuvers. Our opinion is that although provocative maneuvers were very carefully supervised, even non-measurable minor changes in position and intensity of shoulder muscle contractions could result in variable compressive effects. Similarly, we believe that the inconsistent test-retest results were not due to the technique itself, because TcpO2 changes during exercise-induced ischemia are highly reliable and operator-independent (Henni et al., 2018).

Second, we could not confirm the presence or absence of arterial compression using contrast-enhanced imaging in many of the patients. One reason for this was that most patients with positive tests were referred to physical therapy or declined surgery. Radiological imaging in such patients is not indicated. Further, admitting that pallor and coldness did not systematically result from arterial compression, but rather resulted from neurovascular vasoconstriction during nerve compression, indicated that contrast-enhanced imaging techniques should no longer be considered gold-standard techniques. This would be a limitation if our main goal was to evaluate the diagnostic performance of the TcpO2 technique compared to a gold standard. However, this was not our goal. We only intended to test the feasibility and reliability of the technique. Nevertheless, the postoperative results in one of our patients confirmed that DROPmin values improve after surgery.

Third, we only tested the Roos maneuver, although other tests have been proposed to facilitate the diagnosis of TOS. Our choice was deliberate here, and relates to the fact that the Roos maneuver is generally reported as the one that provides a greater number of positive results (Rayan and Jensen, 1995; Nord et al., 2008). Future studies should evaluate the Tcpo2 response to other tests.

Finally, TcpO2 is not considered a routine tool because of the cost of the device and the length of time required to reach stable values, specifically at arm level (Gorska et al., 2015; Chiang et al., 2018). Nevertheless, the diagnosis of TOS should be based on a holistic approach that includes the patient’s history, clinical examination, and minimally invasive investigations. TcpO2 could provide additional non-invasive evidence of abnormal microvascular responses to provocative tests (at least for the Roos test) before radiological imaging is chosen in cases of inefficient physiotherapy.

## Conclusion

In conclusion, to the best of our knowledge, this study is the first to examine microvascular responses during the Roos maneuver in patients with suspected TOS. However, additional studies with a larger number of patients are needed to evaluate the performance of TcpO2 with respect to making the diagnosis of TOS. These studies should compare the test results to a gold standard, analyze the test-retest reproducibility, evaluate the responses to other provocative maneuvers, and estimate its sensitivity to the treatment of neural and/or vascular compression of the outlet. The Roos maneuver should probably be performed at least twice in patients with suspected TOS because of the moderate test-retest reliability of symptoms induced by the Roos test, even during carefully supervised maneuvers. TcpO2 appears to be a promising non-operator-dependent investigative tool that allows for the simultaneous analysis of both arms during dynamic maneuvers in the standing position and investigations of the microvascular response to the Roos test. This might contribute to a holistic approach toward patients with suspected TOS.

## Author Contributions

SH, JH, MA, F-EM, JP, and PA participated in patient recruitment, data acquisition, data analysis, and patient treatment. SH and PA participated in the preparation of the study and provided technical and administrative support for the project. JH, JP, and PA supervised the project. SH, JH, JP, and PA wrote the manuscript. MA and F-EM reviewed and critically revised the draft. All authors approved the final version of the manuscript.

## Conflict of Interest Statement

The authors declare that the research was conducted in the absence of any commercial or financial relationships that could be construed as a potential conflict of interest.
